# Physiatrists’ Attitudes and Knowledge About Cancer Rehabilitation

**DOI:** 10.7759/cureus.28622

**Published:** 2022-08-31

**Authors:** Christian Lopez-Aponte, William Ramos-Guasp, Fernando Sepulveda-Irrizary, Carmen E Lopez-Acevedo, Raul Rosario-Concepcion

**Affiliations:** 1 Physical Medicine and Rehabilitation, University of Puerto Rico, Medical Sciences Campus, Puerto Rico, USA; 2 Physical Medicine and Rehabilitation, Mayo Clinic, Jacksonville, USA

**Keywords:** chemotherapy-related toxicity, perception, cross-sectional studies, neoplasms, rehabilitation

## Abstract

Objective

We aim to assess the awareness and evaluation pattern among physiatrists regarding cancer rehabilitation and associated barriers to access.

Design

The present study is a cross-sectional study in the Physical Medicine and Rehabilitation (PMR) Association Annual Meeting in Puerto Rico that used a 10-item questionnaire to summarize physiatrists’ clinical patterns with their persons diagnosed with cancer (PDWCs).

Results

Thirty-eight (66.7%) participants answered they received minimal to no education about cancer rehabilitation benefits. Cancer patients represented 10% or less of the weekly patient load for 47 (82.5%) physiatrists surveyed. The most common type of cancer encountered was breast cancer for the management of adverse effects. Twenty-nine (50.9%) physiatrists answered that a multifactorial barrier was the cause for limited services within this population group. All participants agreed that rehabilitation is at least sometimes beneficial for cancer patients, and 54 (94.7%) believed these services are needed.

Conclusion

Although rehabilitation specialists learn about the benefits of rehabilitation for PDWCs, there continues to be a limited number of PDWCs evaluated, mainly due to poor access, lack of information about cancer rehabilitation, and economic difficulties. Further efforts should be made to emphasize the importance of integrating rehabilitation techniques in the care of PDWCs.

## Introduction

Few diagnoses in medicine impose a larger physical, emotional, and economic burden than cancer [1]. Overall, cancer represents the second leading cause of death in the USA after cardiovascular diseases [2]. Recent estimates show that almost 17 million people have a history of cancer in the USA, with approximately two million new cases expected in 2021 [3,4]. As medical advances allow for earlier diagnosis and better treatment, cancer survivorship has increased significantly in the last few decades [3]. Likewise, we are now seeing the short- and long-term comorbidities associated with a cancer diagnosis. As such, addressing the unique functional and psychological needs of persons diagnosed with cancer (PDWCs) is paramount to enhance not only their longevity but also their quality of life (QOL).

Cancer rehabilitation entails an impairment-driven multidisciplinary approach aimed at improving the QOL of PDWCs by minimizing the functional and psychological effects of cancer and its treatments. To this end, the role of the physiatrist has been increasingly recognized as essential for the holistic management of PDWCs [5]. Physiatrists are trained to identify, evaluate, and manage the physical and psychological adverse effects that affect the independence and QOL of patients. Unfortunately, studies have shown that only 1%-2% of cancer-associated adverse effects are addressed by rehabilitation specialists [6].

Although the benefits of prehabilitation and rehabilitation have been extensively documented, these services continue to be underpromoted and underutilized by this population [7,8]. Lack of knowledge about the benefits of cancer rehabilitation, socioeconomic barriers to access, and suboptimal adherence have all been identified as contributing factors to limited care [9]. Recently, we found that most oncologists in Puerto Rico receive minimal to no formal education about the benefits of rehabilitation in this population, limiting their referral patterns [10]. We sought to assess the knowledge and perceptions among physiatrists about rehabilitation services for PDWCs.

## Materials and methods

Survey

A group consisting of one oncologist and three Physical Medicine and Rehabilitation (PMR) physicians discussed relevant aspects of cancer rehabilitation to develop a questionnaire that allows the identification of important aspects within cancer rehabilitation education and practice. A questionnaire was developed based on previously published instruments with permission from their authors [11,12]. The questionnaire was reviewed by the department’s research committee, one epidemiologist, and faculty for recommendations. The study was approved by the University of Puerto Rico School of Medicine’s Institutional Review Board before its distribution. The 10-item questionnaire assessed demographic data, experience, educational background, and practice patterns. Demographic questions referred to physician age, sex, type of practice, years since completion of residency, and approximate percentage of cancer patients as part of their caseload seen per week. The initial data also included questions about subjective cancer rehabilitation education and specialty training backgrounds. The physiatrists were asked about their perspectives on cancer rehabilitation, specific cancer diagnoses encountered in practice, and the associated complications for which the patient was referred to the physiatrist. Physicians were asked about specific barriers for patients receiving cancer rehabilitation services and whether they thought cancer rehabilitation was necessary.

Participants

The study took place at the Regional Annual Meeting of the Association of Physical Medicine and Rehabilitation of Puerto Rico in 2017. Physiatrists encountered during the meeting were invited to participate in our study. An informative sheet about the study was delivered to the participants willing to answer the survey where we emphasized to subjects that participation was voluntary and confidential. After the participants verbally agreed to participate, the survey was shared for completion.

Data collection

Questionnaires were collected by the investigators after completion and separated to avoid identification. An identification number was assigned to each participant, and all collected data were kept secure and private using a locked computer at the PMR department at the university where only the investigators were able to access the data. Data was recorded using Microsoft Office Excel 2016 (Microsoft Corp., Redmond, WA, USA).

Statistical analysis

Questionnaire data were analyzed using IBM SPSS for Windows version 25 (IBM SPSS Statistics, Armonk, NY, USA). Characterization of the study group was completed by descriptive analysis. Means and standard deviations (SDs) within the groups were determined for each variable. When survey items allowed participants to provide more than one response, all responses were included for analysis.

## Results

Fifty-seven physiatrists agreed to complete our 10-item survey. Participant demographics are presented in Table 1. The mean (SD) participant age was 49 (13.2) years old, with an even sex distribution. Most surveyed physiatrists reported having practiced the specialty for more than 10 years. More than half practiced primarily in the private setting, while a quarter said their practice was mainly in an academic medical center. The majority (82.5%) indicated that cancer patients represent 10% or less of their weekly patient load.

**Table 1:** Participant demographics SD: standard deviation

Characteristic	Total
Age in years (mean (SD))	49 (13.2)
Sex	
Female	29 (50.9)
Male	28 (49.1)
Years since completing training	
≤5	14 (24.6)
6-10	6 (10.5)
>10	37 (64.9)
Practice setting	
Private or single specialty group clinic	32 (56.1)
Hospital based	7 (12.3)
Academic medical center	2 (3.5)
Multispecialty	14 (24.6)
No answer	2 (3.5)
Training in oncologic rehabilitation	
Advanced	0 (0)
Moderate	18 (31.6)
Minimal	36 (63.2)
None	3 (5.2)
Percentage of cancer patients evaluated per week	
<10%	47 (82.5)
10%	8 (14)
>10%	2 (3.5)
Is rehabilitation beneficial?	
Sometimes	10 (17.5)
Frequently	9 (15.8)
Always	38 (66.7)
Is a cancer rehabilitation program necessary?	
Yes	54 (94.7)
No	3 (5.3)
Would you refer a patient for a cancer rehabilitation program?	
Yes	57 (100)
No	0 (0)

In terms of their training, 18 (31.6%) physiatrists subjectively said they had received moderate education about cancer rehabilitation and its benefits, whereas 38 (66.7%) had received minimal to no education. Forty-two (73.7%) participants answered that the patient’s prognosis did not affect their referral for physical or occupational therapy.

Breast cancer was the most commonly evaluated cancer, followed by brain and spine cancer (Table 2). Thirteen (22.8%) physiatrists answered that they evaluate more than one type of cancer in their clinic. From a functional standpoint, weakness, neuropathic pain, ambulation dysfunction, and prolonged immobilization were the most commonly evaluated disabilities (Table 3).

**Table 2:** Types of cancer evaluated

Cancer type	Physiatrists (number (%))
Breast	23 (40.4)
Multiple	13 (22.8)
Brain	5 (8.8)
Spine	4 (7)
Lung	1 (1.8)
Prostate	1 (1.8)
Gynecologic	1 (1.8)
Neck	1 (1.8)
No response	8 (14)

**Table 3:** Reason to evaluate cancer patients ADL: activity of daily living

Symptom	Physiatrists (number (%))
Lymphedema	15 (26.3)
Dysphagia	7 (12.2)
Weakness	45 (78.9)
Prolonged immobilization	33 (57.9)
ADL difficulties	32 (56.1)
Neuropathic pain	41 (71.9)
Orthosis	10 (17.5)
Osteoarthritis	13 (22.8)
Ambulation difficulties	39 (68.4)
Body image	3 (5.2)
Sexual dysfunctions	3 (5.2)
Amputations	15 (26.3)
Contractures	20 (35.1)
Nociceptive pain	20 (35.1)
Cognitive dysfunctions	5 (8.8)
Pelvic floor dysfunctions	2 (3.5)

Lack of knowledge about the benefits of rehabilitation was identified as the most common barrier to starting a rehabilitation program in this population; however, 29 (50.9%) participants considered this to be a multifactorial problem. All participants agreed that rehabilitation is at least sometimes beneficial for cancer patients, and 54 (94.7%) believed these services are needed. All subjects agreed that, if available, they would refer their patients to a comprehensive cancer rehabilitation program.

## Discussion

To our knowledge, this is the first study describing the knowledge and attitudes toward cancer rehabilitation among general physiatrists in Puerto Rico. Our cohort consisted of 57 physiatrists with a mean age of 49 years, similar to recent national age average estimates [13]. Physiatrists serve an important role in the care of PDWCs. As other studies have established, many people live with cancer as a chronic condition with substantial morbidity and functional disability [7,12]. Physiatrists are in a unique position to evaluate and manage these impairments with the goal of improving functional, psychosocial, and psychoemotional outcomes in this population [12,14].

Fatigue, pain, and impaired mobility are common adverse effects experienced by cancer patients, which could benefit from an evaluation from a physiatrist. Additionally, recent studies have shown that chemotherapy-induced peripheral neuropathy persists in approximately 40% of cancer survivors [2]. Despite the high prevalence of rehabilitation concerns, cancer patients account for less than 10% of the weekly load for most of the surveyed physiatrists. This, along with a stable incidence of approximately 6,000 yearly cancer diagnoses from 2010 to 2017, suggests that most PDWCs in Puerto Rico are not receiving comprehensive care for the musculoskeletal and neurologic adverse effects associated with their diseases [15].

Despite an overlap between the cancer population and other groups within the scope of a general physiatrist, holistic rehabilitation care of cancer patients requires discrete clinical experiences to the breadth of the cancer population and their unique needs. To this end, most of the surveyed physiatrists (66.7%) reported receiving minimal to no formal training during residency about cancer rehabilitation. Considering that a similar percentage (64.9%) of our participants reported having practiced for more than 10 years, future studies evaluating the relationship between years removed from residency and the recent Accreditation Council for Graduate Medical Education (ACGME) requirement that physiatrists demonstrate competence in the rehabilitation and psychosocial care of patients with cancer could provide valuable insight on recent trends and progress [16].

Further, a focused training curriculum in oncology rehabilitation is not standard in most PMR residencies [8]. A study among residency program directors found that cancer rehabilitation education could be improved in both quantity and quality [17]. Likewise, specialized training remains limited to nine fellowships in the USA [18]. This data suggest two important barriers to cancer rehabilitation: (1) current clinical experiences during residency are not sufficient for the general physiatrist to address the unique challenges of the cancer patient, and (2) at the current pace, the output of fellowship-trained cancer rehabilitation subspecialists will not be able to meet the increasing demands for such services. Taken together, these findings highlight the importance of increasing exposure to cancer patients during PMR residency to ensure that general physiatrists attain competence and confidence in the evaluation and management of this population.

Most of the surveyed physiatrists identified a lack of knowledge about its benefits and poor understanding of the referral process as the main barriers to oncology rehabilitation care. To this end, physiatrists must lead the education efforts to address these issues, both among other healthcare providers and in the community. Participating in national oncology forums, offering conferences at local oncology meetings, and participating in routine multidisciplinary meetings are all initiatives that could increase the overall awareness of the physical and psychosocial benefits of cancer rehabilitation.

Most rehabilitation programs establish goals based on functional status prior to diagnosis, stage of disease, and prognosis. In our study, all of the surveyed physiatrists consider cancer rehabilitation beneficial, regardless of the patient’s disease stage or prognosis. Other studies have shown that only 35% of physiatrists accepted cancer patients regardless of their prognosis [11]. Evidence has shown that patients benefit from rehabilitation care throughout all stages of the disease and that, as cancer advances, physical training and rehabilitation improve performance in daily life, psychosocial status, and QOL [19]. Likewise, recent studies showing the benefits of an intensive rehabilitation program for PDWCs awaiting surgical intervention provide further evidence of the value of a comprehensive rehabilitation program, either at home or at a specialized center [20,21]. When deemed necessary, an inpatient rehabilitation program may allow for a multidisciplinary and comprehensive approach to managing these limitations [22].

Our study represents a small cohort limited to the physiatrists community in Puerto Rico, which limits the generalizability of our results. Future studies aimed at identifying barriers in specific geographic areas and socioeconomic circumstances may allow for a targeted local and national response. Recall bias is another possible limitation given the design of our study.

## Conclusions

Rehabilitation care is needed but currently is suboptimal to meet the growing demand of PDWCs. Unfortunately, most patients are not routinely referred to physiatrists for evaluation of musculoskeletal and neurologic adverse effects. Although most physiatrists acknowledge the value of rehabilitation care for this population, multiple barriers that affect its delivery exist. Some of these barriers include limited exposure to the cancer population during residency, lack of knowledge of its benefits, limited access to rehabilitation services, and the need for improvement in referral mechanisms.
